# Caregiver-Assisted Pain Coping Skills Training for Patients With Dementia: A Pilot Study

**DOI:** 10.1093/geroni/igab046.1119

**Published:** 2021-12-17

**Authors:** Laura Porter, Francis Keefe, Deborah Barnes, Lisa Gwyther, Kenneth Schmader, Christine Ritchie

**Affiliations:** 1 Duke University Medical Center, Durham, North Carolina, United States; 2 Duke University School of Medicine, Durham, North Carolina, United States; 3 University of California, San Francisco, San Francisco, California, United States; 4 Duke Center for Aging, Chapel Hill, North Carolina, United States; 5 Duke, Durham, North Carolina, United States; 6 Massachusetts General Hospital, Boston, Massachusetts, United States

## Abstract

Pain is common and undertreated in patients with dementia, and contributes to disability, psychological distress, neuropsychiatric symptoms and caregiver stress. The goal of this study was to develop a caregiver-assisted pain coping skills training protocol tailored for community-dwelling adults with mild-moderate dementia and their family caregivers. We conducted interviews with patients and caregivers to develop the protocol. We then conducted a single arm pilot test of the intervention’s feasibility and acceptability. Patients were recruited from an outpatient memory care clinic and screened for pain using the validated Pain, Enjoyment, General Activity (PEG) scale. The intervention included five sessions of training in pain assessment, relaxation, pleasant activity scheduling, and integrative movement. Initially sessions were conducted in person or by videoconference according to the dyad’s preference; during COVID-19 (latter half of study) all sessions were conducted remotely. Eleven dyads consented and provided baseline data [patients: mean age=77.7 years (SD=4.8), 70% non-Hispanic white; caregivers: mean age=69.6 years (SD=13.3); 91% non-Hispanic white; 73% spouses]. Nine dyads (82%) completed all five sessions. Caregivers reported high levels of satisfaction with the intervention (mean=3.4 on 1-4 scale) and frequent use of pain coping skills (mean=3-4 days/week). On average, patients reported pre-post decreases in pain severity (mean=-1.2, SD=1.8) and pain interference (mean=-0.64, SD=0.67) on the Brief Pain Inventory. Overall these findings suggest that a behavioral pain coping intervention for patients with mild-moderate dementia and their caregivers is feasible, acceptable, and potentially helpful for managing pain.

